# Comparison of the effect of preoperative multimedia video information on preoperative anxiety in patients undergoing procedures under central neuraxial block: a prospective, single-blind, randomized controlled study

**DOI:** 10.4314/ahs.v26i1.8

**Published:** 2026-03

**Authors:** K Duraiyarasan, Ruchi Kumari, Anshul Goyal, Munisha Agarwal, Neelam Prasad

**Affiliations:** Anaesthesiology and Critical Care, Maulana Azad Medical College and associated Lok Nayak Hospital, New Delhi

**Keywords:** Preoperative anxiety, Multimedia video, State Trait Anxiety Inventory

## Abstract

**Background and Aims:**

Preoperative anxiety is one of the most common perioperative complications observed in patients undergoing surgery. The use of State Trait Anxiety Inventory (STAI) and Visual Analogue Scale (VAS-A) scores for anxiety has been demonstrated to reduce the preoperative anxiety levels. We evaluated the effect of preoperative multimedia video information on preoperative anxiety and haemodynamic parameters in patients undergoing procedures under central neuraxial block (CNB).

**Materials and Methods:**

Sixty patients of either sex, aged 18-60 years undergoing elective surgeries under CNB were randomized into Group V (video) and Group NV (non-video). The primary outcomes included STAI score and VAS-A score assessed one hour prior to surgery. The secondary outcomes involved haemodynamic parameters before performing CNB and, patient's cooperation for CNB assessed by three-point scoring system.

**Results:**

Demographic parameters were comparable in both groups. The HR, SBP, DBP and MAP showed a significant increase from their baseline values before surgery in Group NV as compared to Group V (p<0.001). In Group V, STAI scores decreased significantly (p<0.001), while Group NV showed a significant increase (p<0.001). VAS-A scores for anxiety decreased in Group V and increased in Group NV before surgery (p<0.001). Group V demonstrated higher cooperation scores (score ≥2).

**Conclusion:**

Preoperative multimedia information was associated with reduced anxiety, better haemodynamic stability, and improved patient cooperation. Employing multimedia information in the form of video may prove beneficial for allaying preoperative anxiety in adults undergoing surgery under CNB.

## Introduction

Preoperative anxiety is one of the most common perioperative complications observed in a substantial proportion of adults undergoing surgery, ranging from approximately 11% to 80%^1^. A lack of knowledge about operating theatre environment, surgical procedures, and anaesthetic techniques may be the underlying cause^2^. It often triggers cognitive, emotional, and physiological responses^3^. Patients with extreme preoperative anxiety, tend to require larger doses of induction agents and analgesics and have longer hospital stays^4^. Various methods have been tried to reduce patient's anxiety, including written pamphlets and questionnaires. Multimedia information has also been tried successfully to allay patient's anxiety^5^. Studies have shown that visual information can enhance patient comprehension and improve the consent process for medical procedures, thereby reducing anxiety^6^. The use of State Trait Anxiety Inventory (STAI) score and Visual Analogue Scale score for anxiety (VAS-A) to quantify the effects of preoperative assessment by an anaesthesiologist has demonstrated a reduction in patient's anxiety^7^. The STAI score consists of self-reported scales for measuring patient's anxiety, ranging from 6 (minimum) to 24 (maximum), with categories of 6-12 (low or no anxiety), 13-18 (moderate anxiety) and, 19-24 (high anxiety). The VAS score is a scale of 0-100 mm, where 0 represents “no anxiety” and 100 signifies “maximum anxiety”. The combination of an anaesthetic pre-assessment and a preoperative multimedia video can effectively reduce anxiety in patients by promoting positive expectations and providing emotional support. Therefore, we conducted this study to evaluate the effect of preoperative multimedia video information on both preoperative anxiety and haemodynamic parameters in patients undergoing procedures under central neuraxial blocks (CNB).

## Materials and methods

This prospective, randomized, single-blind study was conducted from March 2018 to March 2020 after Institutional Review board approval [F. No. 17/IEC/MAMC/2017/ANAES/06] [CTRI/2018/05/013737]. Written informed consent was obtained from all the participants. We enrolled sixty ASA I/II patients of either sex, aged 18-60 years undergoing elective surgeries under CNB. The primary outcomes included the patient's anxiety score using State Trait Anxiety Inventory (STAI) score and Visual Analogue Scale (VAS) score assessed one hour prior to surgery. The secondary outcomes involved haemodynamic parameters i.e., heart rate (HR), systolic (SBP), diastolic (DBP) and, mean arterial pressure (MAP) assessed before performing CNB. Additionally, patient's cooperation for CNB assessed by a three-point scoring system, ranging from 0-2; 0 (not cooperative), 1 (moderately cooperative) and, 2 (very cooperative). Patients with significant cardiovascular, neurological and psychiatric illnesses were excluded. Patients were randomized using computerized randomization into two groups i.e., Group V (video group, n=30) and Group NV (non-video group, n=30). Group V was shown a small video of the CNB procedure one day prior to surgery, while no video was shown to the Group NV; instead, they received a routine verbal explanation of the procedure. Group allocations were sealed in consecutively numbered opaque envelopes, prepared by an anaesthesiologist who was not involved in the study. Throughout the study period, assessor (A1) remained blinded to the group allotted to the patient. The showing of the video to patients in Group V was conducted by an independent anaesthesiologist (A2) who did not participate further in the study. The anaesthesiologist (A3) who carried out the CNB was blinded to patient's group allocation. One day prior to surgery, the preoperative anxiety scoring system was explained to the patient in their own language by anaesthesiologist (A1), and haemodynamic parameters such as HR, SBP, DBP and MAP were assessed. Thereafter, the baseline anxiety was assessed using STAI and VAS-A scores for each patient. Each patient received a 0.25 mg tablet of alprazolam the night before surgery. On the day of surgery, the STAI score and VAS-A score were re-assessed one hour before surgery in the preoperative area by A1. On arrival in the operation theatre, ASA standard monitoring devices such as pulse oximeter, NIBP, and ECG were attached. After five minutes, haemodynamic parameters i.e., HR, SBP, DBP and MAP were noted. The patient was then positioned for CNB, and the block was performed by A3. After completion of the procedure, the same anaesthesiologist assessed the patient's cooperation for the CNB on a three-point scoring system. Based on a previous investigation^5^, we determined that a sample size of 30 patients in each group is needed to detect a clinically significant difference in anxiety scores, given a type I error of 5% and a study power of 80%. The quantitative variables were expressed as mean ± SD and compared between groups using the unpaired t-test, and within each group at various follow ups using the paired t-test. Qualitative variables were reported as frequencies or percentages and compared using the Chi-square test. A p-value < 0.05 was considered statistically significant. Statistical analysis was performed using SPSS version 25.0.

## Results

The demographic parameters were comparable in both groups [Table 1]. The baseline anxiety score (STAI) in females (15.55±1.92) was slightly higher as compared to males (14.94±1.57) but this was not statistically significant (p=0.27). Forty percent patients in Group V were uneducated while in Group NV the percentage was 53.3. However, the STAI score for degree holders (14.86±1.41) did not significantly differ from that of less educated patients (p=0.632). Similarly, the STAI score was 14.68±1.60 in patients with previous history of surgery under CNB in comparison to those who were unexposed to any surgery 15.22±1.65, and this difference was not statistically significant (p= 0.243).

**Table 1:** Mean distribution of various patient characteristics among both groups

Characteristics	V (mean±SD)	NV (mean±SD)	p-value (V vs NV)
**Age (years)**	34.80±14.83	37.67±11.6	0.204
**Weight (kg)**	63.13±14.83	69.53±9.06	0.003
**Height (cms)**	163.63±8.21	167.87±6.07	0.013
**BMI (kg/m^2^)**	23.55±2.41	24.59±2.17	0.042

With regards to haemodynamic parameters, Group V showed a significant decrease in heart rate (77.67±6.28) before surgery compared to the baseline (82.93±7.23), whereas Group NV showed an increase in heart rate (83.27±7.13) before surgery in comparison to the base line (77.40±6.69) (p<0.001). This observed difference was statistically highly significant [Table 2].

**Table 2:** Distribution of HR, SBP, DBP and MAP among both the groups before and after intervention

Variables	HR (V)	HR (NV)	SBP (V)	SBP (NV)	DBP (V)	DBP (NV)	MAP (V)	MAP (NV)
**Baseline** (mean±SD)	82.93±7.23	77.40±6.69	128.13±11.36	123.33±11.56	81.40±7.93	78.60±5.97	96.63±8.52	93.27±7.19
**Before surgery** (mean±SD)	77.67±6.28	83.27±7.13	123.53±9.71	131.67±6.91	77.87±5.51	84.40±4.65	92.73±6.6	99.67±4.84
**p-value**	<0.001	<0.001	0.004	<0.001	0.003	<0.001	0.001	<0.001

In Group NV, the SBP, DBP and MAP showed a substantial increase from their respective baseline values before surgery. In contrast, Group V displayed a significant decrease in these parameters, and these differences were statistically highly significant (Group NV: SBP, p<0.001; DBP, p<0.001; MAP, p<0.001; Group V: SBP, p=0.004; DBP, p=0.003; MAP, p=0.001) [Table 2]. In Group V, there was a notable reduction in the STAI score before surgery (14.57±1.3) from the baseline (15.70±1.6), with a p-value of <0.001 whereas, in Group NV, there was a significant increase in the STAI score (15.40±1.4) from the baseline (4.40±1.43) before surgery, and this difference was statistically highly significant (p<0.001) [Table 3].

**Table 3:** Distribution of STAI and VAS-A scores among both groups before and after the intervention

Variables	STAI (V)	STAI (NV)	VAS-A (V)	VAS-A (NV)
**Baseline** (mean±SD)	15.70±1.6	14.40±1.43	5.90±1.49	4.73±1.28
**Before surgery** (mean±SD	14.57±1.3	15.40±1.4	5.20±1	5.77±1.01
**p-value**	<0.001	<0.001	0.003	<0.001

The VAS-A score before surgery (5.20±1) significantly decreased from the baseline (5.90±1.49) in Group V, with a p-value of <0.001. In contrast, Group NV showed a significant increase (5.77±1.01) from the baseline (4.73±1.28) and, this difference was statistically highly significant (p<0.001) [Table 3]. Regarding the patient's cooperation score, Group V were more co-operative (score≥2) as compared to Group NV [Table 4].

**Table 4:** Distribution of cooperation score among both groups

Cooperation score	V (n, %)	NV (n, %)	p-value (V vs NV)
**0**	0	10 (33.33%)	<0.001
**1**	19 (63.33%)	17 (56.67%)	0.299
**2**	11 (36.67%)	3 (10%)	0.007
**Total**	30 (100%)	30 (100%)	-

## Discussion

There was no difference in the demographic parameters in both study groups. However, it was observed that patients with higher BMI tended to have elevated pre-operative anxiety scores. This may be attributed to their heightened perception of surgical and anaesthetic risks, such as wound infection, delayed healing, challenges in airway management, complexities in drug dosing, and concerns related to body image. Our study included both literate and illiterate patients in contrast to the study done by Dias R et al, where all participants were literate and could read and complete the questionnaire on their own in English, Hindi, or the local language (Marathi)^5^. We used an additional tool i.e., VAS-A score to assess pre-operative anxiety so that those who could not understand the STAI scoring system adequatelywould have no problem marking their preoperative anxiety levels on the VAS scale.

Our study revealed that there was a significant decline in STAI scores from the baseline just before surgery with a p-value of <0.001, among patients who were shown multimedia video in comparison to patients who received verbal information about the CNB procedure. This finding is concurrent with the study conducted by Dias R et al, where patients who were not shown a multimedia information film experienced a significant increase in STAI scores from baseline values before surgery^5^.

Similarly, Jlala et al demonstrated that the control group (patients who did not watch the film) exhibited a significant increase in STAI scores from baseline immediately before surgery (p<0.001). However, the VAS-A score did not differ significantly between the video and non-video groups before surgery. This lack of significant difference was attributed to the central tendency bias, wherein patients refrained from using extreme scores when uncertain about how to respond. This tendency was due to unfamiliarity with the assessment method for anxiety. It also showed that the STAI score is better at detecting even subtle changes in anxiety^8^. Our results are also consistent with those of Kiran et al, whose study involving 200 patients undergoing cataract surgery showed that preoperative video significantly reduced patients' preoperative anxiety^9^. In our study, there was a noteworthy reduction in HR, SBP, DBP and, MAP from the baseline before surgery. This observed decline could be the result of the reduction in preoperative anxiety by multimedia video information. The decrease in anxiety inhibits the hypothalamic-pituitary release of adrenocorticotrophic hormone and cortisol into the blood stream, thereby reducing the activation of sympathetic nervous system^10^. We did not observe any episodes of vasovagal response, hypotension, or bradycardia, which may be attributed to appropriate intravenous fluid administration prior to the block, proper spinal drug dosing, and optimal patient positioning during the procedure. We also used the cooperation score to assess the effect of preoperative informative or educational videos about CNB on patients' willingness to cooperate during the procedure. We found that a higher percentage of patients who were shown the video were more cooperative (36.67%) as compared to those who were not (10%). Conversely, a significant proportion of patients who received only verbal information were found to be non-cooperative (33.33%) for the procedure. Assessment of the patient's cooperation with the procedure allowed us to examine the potentially significant effects of knowledge acquisition and retention resulting from the video, particularly in terms of anxiety reduction. The cooperation score reflected the amount of knowledge retained by the patient about the procedure. Visual stimuli cause a faster and stronger reaction as compared to verbal delivery of any information. They help the users to engage with the content and, thereby influencing the degree of retention of information. Visual memory, known for its superiority over verbal memory, has the tendency to persist for a longer duration^11^.

The degree to which each patient manifests anxiety is influenced by various factors, such as the patient's susceptibility to preoperative anxiety, age, gender, current health status, educational status, past surgical experiences, nature and extent of the proposed surgery, and socioeconomic status. Females, younger patients, and those without prior history of surgical experience tend to have higher levels of preoperative anxiety^12^-^14^. Our study observed a higher mean STAI score in females as compared to males, although it was statistically insignificant. This finding is in agreement with the results reported by Dias et al and Jlala et al, both of whom showed that women tend to have a higher baseline anxiety score compared to men. The increased anxiety in women is attributed to their heightened sensitivity to fearful events and hormonal fluctuations^5^,^8^. In addition, females are considered to be more expressive in conveying their anxiety in comparison to males^11^. However, its worth noting that Kiyohara LY et al showed no significant difference in state anxiety levels between males and females^15^.

The pervasive “fear of the unknown” is a major contributor to preoperative anxiety. Educated people can comprehend and understand the information about the anaesthetic procedure to be undertaken more effectively than those with less education. We compared the baseline anxiety score of the patients based on educational status which showed that the baseline anxiety score was less in patients who were graduates than patients who were less educated, although this difference was not statistically significant. This observation is consistent with the study conducted by Serkan Tulgar et al, who showed that STAI scores were lower in university graduates than those who were merely literate (p <0.05)^16^. It's noteworthy that we did not find any statistically significant difference in our study, probably due to a smaller number of patients who had higher levels of education^14^ (graduates out of 60) as compared to those in their study (54 graduates out of 330). However, Seifu Nigussie et al reported a different trend, indicating that the level of preoperative anxiety increases with an increased level of education, as educated patients tend to be more aware of complications related to anaesthesia and surgery^17^. In our study, we observed that patients having previous history of surgery under CNB were less anxious than patients who had not undergone any surgery before. Although, this difference was not statistically significant (p=0.243), possibly due to the smaller size of our study population. However, this finding is in contrast with Serkan Tulgar et al, who found a statistically significant difference (p<0.01)^16^. This decrease in preoperative anxiety in these patients could be attributed to a reduced fear of surgical procedure and more understanding of anaesthesia and surgery^3^.

## Conclusion

Based on the aforementioned findings, we concluded that multimedia information about CNB, in the form of video, is a simple, easily accessible, safe and efficient non-pharmacological method of allaying preoperative anxiety in adult patients. This not only contributes in reducing the patient's anxiety but also aids in better control of ensuing haemodynamic parameters. Furthermore, it enhances patient cooperation during performance of CNB. Hence, we strongly recommend that multimedia video information should be routinely used as a standard method for allaying preoperative anxiety in adults undergoing surgery under CNB.
